# Trends in malaria research in 11 Asian Pacific countries: an analysis of peer-reviewed publications over two decades

**DOI:** 10.1186/1475-2875-10-131

**Published:** 2011-05-18

**Authors:** Finn Andersen, Nick M Douglas, Dorina Bustos, Gawrie Galappaththy, Gao Qi, Michelle S Hsiang, Rita Kusriastuti, Kamini Mendis, George Taleo, Maxine Whittaker, Ric N Price, Lorenz von Seidlein

**Affiliations:** 1 Global Health Division, Menzies School of Health Research, PO Box 41096, Casuarina, NT 0811, Australia; 2 Centre for Tropical Medicine, Nuffield Department of Clinical Medicine, University of Oxford, UK; 3 Research Institute for Tropical Medicine, Department of Health, FICC, Alabang, Muntinlupa City, Philippines; 4 National Malaria Control Programme, Ministry of Health, Colombo, Sri Lanka; 5 Jiangsu Institute of Parasitic Diseases, Wuxi, Jiangsu, 214064, P.R.China; 6 Global Health Group, University of California, San Francisco, USA; 7 Vector Borne Disease Control Programme, Ministry of Health, Jakarta, Republic of Indonesia; 8 World Health Organization, Geneva, Switzerland; 9 Malaria & Vector Borne Diseases Control Programme, Ministry of Health, Vanuatu; 10 Australian Centre for International and Tropical Health, School of Population Health, The University of Queensland, Herston, Australia

## Abstract

**Background:**

Quantitative data are lacking on published malaria research. The purpose of the study is to characterize trends in malaria-related literature from 1990 to 2009 in 11 Asian-Pacific countries that are committed to malaria elimination as a national goal.

**Methods:**

A systematic search was conducted for articles published from January 1990 to December 2009 in PubMed/MEDLINE using terms for malaria and 11 target countries (Bhutan, China, North Korea, Indonesia, Malaysia, Philippines, Solomon Islands, South Korea, Sri Lanka, Thailand and Vanuatu). The references were collated and categorized according to subject, *Plasmodium *species, and whether they contained original or derivative data.

**Results:**

2,700 articles published between 1990 and 2009 related to malaria in the target countries. The annual output of malaria-related papers increased linearly whereas the overall biomedical output from these countries grew exponentially. The percentage of malaria-related publications was nearly 3% (111/3741) of all biomedical publications in 1992 and decreased to less than 1% (118/12171; p < 0.001) in 2009. Thailand had the highest absolute output of malaria-related papers (n = 1211), followed by China (n = 609) and Indonesia (n = 346). Solomon Islands and Vanuatu had lower absolute numbers of publications, but both countries had the highest number of publications per capita (1.3 and 2.5 papers/1,000 population). The largest percentage of papers concerned the epidemiology and control of malaria (53%) followed by studies of drugs and drug resistance (47%). There was an increase in the proportion of articles relating to epidemiology, entomology, biology, molecular biology, pathophysiology and diagnostics from the first to the second decade, whereas the percentage of papers on drugs, clinical aspects of malaria, immunology, and social sciences decreased.

**Conclusions:**

The proportion of malaria-related publications out of the overall biomedical output from the 11 target Asian-Pacific countries is decreasing. The discovery and evaluation of new, safe and effective drugs and vaccines is paramount. In addition the elimination of malaria will require operational research to implement and scale up interventions.

## Background

Research and development play a major role in many enterprises. A typical ratio of research and development for an industrial company is about 3.5% of revenues while in some pharmaceutical companies this ratio may be several-fold higher [1]. Although malaria control and elimination is not an industrial enterprise, most experts would agree that efforts supported by evidence and new tools are more likely to be successful. Very little quantitative data have been published to quantify the malaria research effort particularly in the malaria endemic countries of Asia. The lack of knowledge of research outputs reflects the intrinsic difficulties in quantifying these measures. The number of publications is an indirect measure of scientific research outputs and activities. Although not all research results in a publication, there is a broad consensus that scientific publications reflect the research effort and funding opportunities.

There is uncertainty about what tools are needed and how tools should be used in order to achieve elimination. In this context research and development will play a pivotal role in shaping future malaria elimination activities. Much can be learned from successful programmes, but the heterogeneity of the malaria epidemiology between nations makes it essential to adapt programmes to the current local needs. In 2009, 11 countries in the Asian Pacific region announced national goals to eliminate malaria (Bhutan, China, North Korea, Indonesia, Malaysia, Philippines, Solomon Islands, South Korea, Sri Lanka, Thailand and Vanuatu), and together established the Asian Pacific Malaria Elimination Network (http://apmen.org/). To develop a better understanding of the malaria research outputs and activities in these APMEN countries, the number of peer-reviewed publications has been reviewed, with the purpose of characterizing trends in malaria-related literature over the last two decades.

## Methods

### The target countries

The 11 APMEN countries have made significant progress towards malaria elimination on a national or sub national level, and have made an official commitment toward elimination. The population of the countries included in this study varied greatly, ranging from 246,000 people in Vanuatu to 1,381,796,000 in China (Table 1)[2]. Gross domestic product (GDP) per capita varied from USD 16,700 in South Korea to USD 1,200 in the Solomon Islands [2]. The population at risk of vivax malaria ranged from 461,951,000 in China to 242,000 in Vanuatu [3]. The largest population at risk of falciparum malaria was 132,878,000, found in Indonesia [4,5].

**Table 1:** Key demographic and economic data from the 11 APMEN countries

	**Population/1000**[2]	**GDP **[2]**(billion USD)**	**GDP per capita **[2]**(thousand USD)**	**Health Expenditure per capita **[14]**(USD)**	**HDI**[15]	**PAR PF/1000 **[5]	**PAR PV/1000 **[3]	**reported Pf cases 2009 **[16]	**reported Pv cases 2009**[16]
Bhutan	723	1.3	1.8	49	0.62	411	481	559	413
China	1,381,796	4909.0	3.7	94	0.66	16,646	461,951	948	8,214
Indonesia	232,544	539.4	2.3	41.8	0.60	132,878	129,618	212,501	237,929
Korea North	23,963	27.8	1.2	14	NA	0	22,079	0	14,845
Korea South	48,517	832.5	16.7	1168	0.88	0	3,022	0	1,343
Malaysia	27,949	191.5	6.8	259	0.74	27,845	27,902	1,885	3,379
Philippines	93,617	161.0	1.7	52	0.64	50,154	50,339	13,933	4,951
Solomon Islands	536	0.7	1.2	44	0.49	534	535	19,580	8,544
Sri Lanka	20,410	41.3	2.0	62	0.66	10,231	11,986	29	529
Thailand	68,141	263.9	3.9	136	0.65	40,839	41,966	9,486	13,616
Vanuatu	246	0.6	2.6	67	0.69	242	242	1,543	1,618

NA = not available, USD = United States Dollars, HDI = Human Development Index, PAR = population at risk, PF = *Plasmodium falciparum*, PV = *Plasmodium vivax*

### Criteria for considering articles for this review

Articles with an abstract in English language relating to malaria in the 11 target countries within the time frame of January 1990 to December 2009 were included in the study. The search methods for acquiring article references are described in supplementary materials (Additional files 1, 2 and 3).

### Processing reference library

The publication type for each article was derived from the PubMed/Medline database and articles were categorized as containing original data, or non-original, derivative data or being irrelevant to malaria research based on these publication types (see Additional file 2). References not provided with a publication type were manually tagged. Each paper was assigned to at least one of the following subjects based on the keywords included in the reference: Diagnostics, Molecular/Genetics, Clinical, Entomology/Insecticides, Biology/Biochemistry, Social Sciences/Health Policy, Epidemiology/Control, Immunology/Vaccines, Pathophysiology, Drugs/Drug resistance [6]. The search algorithm used to categorize the papers is shown in Additional file 3.

All papers were further classified as relating to *Plasmodium falciparum *or *P. vivax *depending on whether the term 'falciparum' or 'vivax' appeared in the title, abstract or keywords. Malaria-related papers in which the *Plasmodium *species was not defined were categorized as "neither". These papers were manually reviewed to assess their relevance for malaria and excluded if they were not sufficiently relevant. Articles were also classified as describing clinical trials if their publication type field contained 'clinical' or 'randomized controlled'.

The total number of overall biomedical publications from the target countries were gathered by removing the keywords for malaria (*P. falciparum, P. vivax*) from the search algorithm.

### Statistics

Data were processed using Excel (Microsoft, WA, USA) filters and Excel was also used to compute the correlation coefficient r^2^. Statistical comparisons were computed with Statcalc (EpiInfo, version 6, Centers for Disease Control and Prevention, Atlanta, USA). For categorical variables, percentages and corresponding 95% confidence intervals (95% CI) were calculated using Wilson's method. Proportions were examined using χ^2 ^with Yates' correction.

## Results

**Figure 1:**
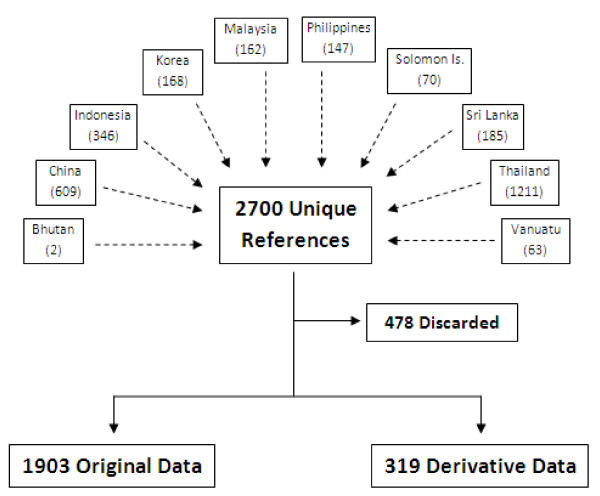
**Assembly of references**.

In total, 2,700 unique malaria-related, peer-reviewed references published between 1990 and 2009 were identified as associated with the 11 target countries. Of these papers 1,903 (70%) were articles containing original data and 319 (12%) were articles containing derivative data. The remaining papers (478; 18%) were found to be insufficiently related to malaria. The assembly of references by country and subjects is shown in Figure 1.

**Figure 2:**
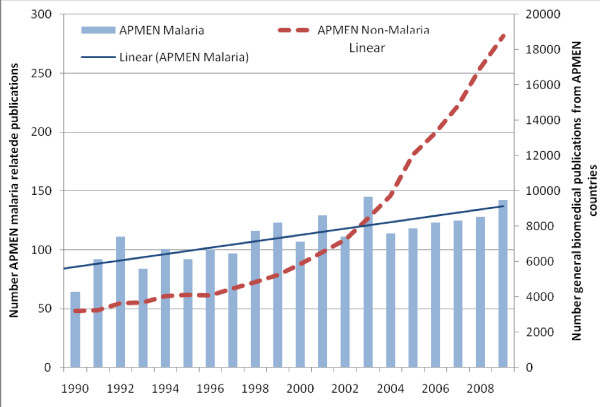
**The yearly number of malaria-related and all biomedical publications other than malaria from the 11 target countries, 1990 to 2009**.

**Figure 3:**
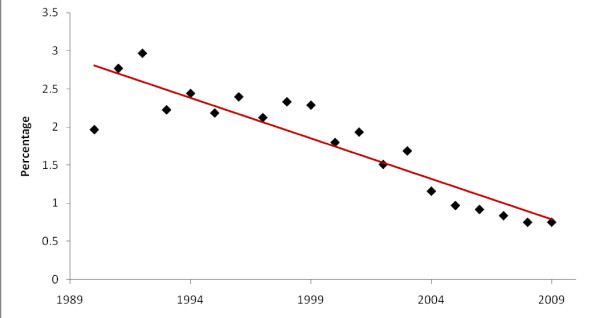
**The percentage of malaria-related out of total biomedical papers by year of publication**.

To visualize general trends in peer reviewed publications, the number of malaria-related publications was graphed and compared with overall biomedical publications over time (Figure 2). Over the 20-year study period, the number of malaria-related publications rose 2.1 fold in a linear fashion (linear trendline with r^2 ^= 0.66), from 64 per year in 1990 to 136 per year in 2009. In the first decade of analysis 980 malaria-related publications were identified, increasing to 1,242 during the following decade. The proportion of papers reporting original data remained relatively stable throughout the two decades at around 85%. Over the same period the number of biomedical publications relating to the target countries increased exponentially (exponential trendline with r^2 ^= 0.95). The total number of biomedical publications increased from 40,567 in the decade from 1990 through 1999 to 113,714 in the decade from 2000 through 2009. The percentage of malaria-related publications was nearly 3% (111/3,741) of all biomedical publications in 1992 and decreased yearly to less than 1% (118/12,171) by 2005, staying below 1% thereafter (p < 0.001; Figure 3).

### Publication output by country

**Figure 4:**
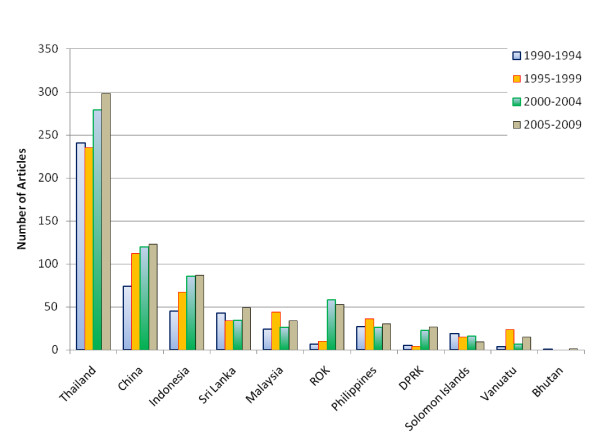
**The number of malaria-related articles in each country per 5 year period**.

Thailand had the highest output of malaria-related papers (n = 1,211), followed by China (n = 609) and Indonesia (n = 346). The output of malaria-related publications for Thailand, China, Indonesia, South Korea and North Korea increased steadily (Figure 4). Following the re-emergence of vivax malaria in 1993, North and South Korea experienced the greatest relative increase in publications from the first decade to the second (512% and 422% respectively). Although the Solomon Islands and Vanuatu had some of the lowest absolute numbers of publications, they had the highest number of publications per capita (1.3 and 2.5 papers/1000 population). Only two malaria-related papers during the 20-year period were associated with Bhutan [7,8]. There was a weak correlation between the size of the population at risk of malaria in a country and the numbers of papers on malaria-related to the country (r^2 ^= 0.13). There was no correlation between the GDP per capita, human development index, health expenditure per capita and the number of publications from a country.

### Publication output by subject of publication

**Figure 5:**
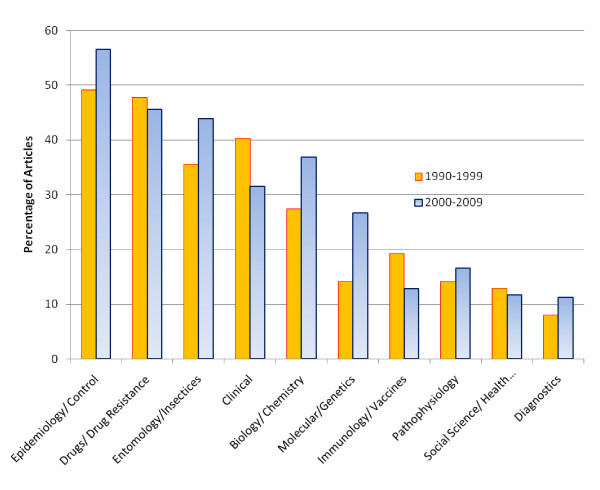
**Percentage of malaria-related articles by topic, 1990-1999 and 2000-2009**.

Publications were categorized into 10 subjects and charted for the first and second decades of the study; Figure 5. The epidemiology and control of malaria accounted for 53% (1,183/2,222) of publications, followed by drugs and drug resistance (47% 1,032/2,222; p < 0.001)). Between the first and second decade there was an increase in the proportion of articles on epidemiology (1.15 fold; p < 0.001), entomology (1.24 fold; p < 0.001), biology, molecular biology, pathophysiology and diagnostics. In contrast there was a decrease in the proportion of papers on immunology (0.67; p < 0.001), clinical aspects of malaria (0.78 fold; p < 0.001), drugs (0.95 fold; p = 0.3), and social sciences (0.91; p = 0.4).

### Publication output by parasite species

**Figure 6:**
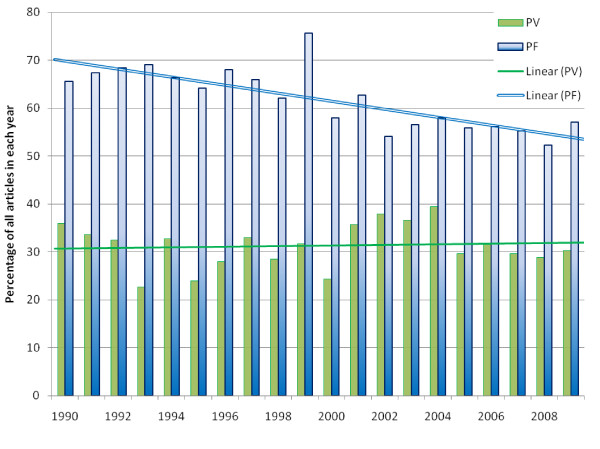
**Comparison of the yearly percentage of articles on Plasmodium vivax (PV) and falciparum (PF), 1990 to 2009 (with trendlines)**.

**Figure 7:**
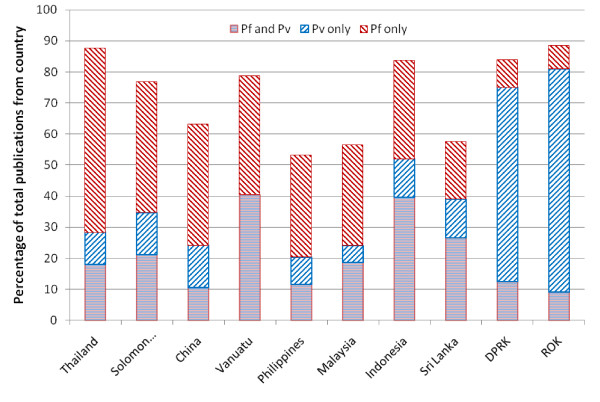
Percentage of articles relating to Plasmodium vivax (Pv), falciparum (Pf) or both species by country*. *ROK = Republic of Korea, DRRK = Democratic People's Republic of Korea. Bhutan provided 2 data points and is not shown.

The proportions of papers on *P. vivax *and *P. falciparum *are shown in Figure 6. The proportion of all articles associated with *P. falciparum *decreased from 67% (661/980) in the first decade to 56% (703/1242) in the second (p < 0.001). In contrast, the proportion of articles associated with *P. vivax *fluctuated between 22% and 38%, with no major change from the first decade (30%, 296/980) to the second (32%, 403/1242; p = 0.5). In only two countries (North Korea 89%; 42/47 and South Korea 81%; 98/121) did the majority of malaria-related papers deal with *P. vivax *and not with *P. falciparum*. Malaria-related articles not explicitly relating to either species increased from 22% (215/980) in the 1990's to an average of 29% (361/1242) in the second decade (p < 0.001). This group of publications mostly included papers on molecular mechanisms, entomology and insecticides but also the less frequently encountered human *Plasmodium *sp. (ovale, malariae, knowlesi). Overall there were more publications on *P. falciparum *compared with *P. vivax (*Figure 7), with the total ratio being 1.95 *P. falciparum *related articles for every *P. vivax *article (95%CI 1.89 to 2.00). The trend of *P. falciparum *articles being dominant was apparent within each subject, with the exception of Social Sciences/Health Policy, where the proportion of articles that did not relate specifically to either *Plasmodium *species was higher than the proportion that did. The majority of articles describing clinical trials related to *P. falciparum *(85%; 260/307). But the proportion of publications describing clinical trials relating to *P. falciparum *decreased steadily over the two decades from 89% (62/70) between 1990 and 1995 to 81% (52/64) between 2005 and 2009 (p = 0.2) In contrast the percentage of publications describing clinical studies relating to *P. vivax *or neither species remained stable.

## Discussion

Malaria related publications from 11 Asian Pacific countries committed to malaria elimination increased in a linear fashion over the last two decades, doubling between 1990 and 2009. In contrast the overall biomedical output grew exponentially. This exponential growth has largely been driven by China, which increased its research output dramatically over the study period. Funding for global malaria research has increased substantially during the study period [9], yet this is not reflected in the number of malaria-related publications in APMEN countries. Over two decades, there has been a three-fold reduction in the proportion publications from the APMEN countries related to malaria; currently less than one percent of biomedical research is related to malaria. The reasons for this are likely to be complex. Potential explanations include the perceived decreased importance of malaria as its burden in the APMEN countries falls with successful control efforts. Simultaneously other areas of medical research, such as cancer have been growing rapidly with the changing economy and demography. Whilst the funding for malaria has increased in absolute terms, the funding for other medical disciplines has grown even faster and is now out-competing malaria research.

The research output per capita varied considerably between countries. Surprisingly no significant correlation could be detected between population at risk, GDP per capita, human development index, health expenditure per capita and the number of publications per capita. Other local factors are likely to play an important role in productivity. In Thailand for instance, one research unit produced over 800 publications on malaria over the last 20 years and has thus ensured the prominence of Thailand in terms of publication outputs. The proportion of research output specifically related to *P. falciparum *decreased whereas the proportion of articles relating to *P. vivax *remained relatively stable. This finding may reflect a healthy correction of research priorities as *P. vivax *creates a large proportion of malaria related morbidity in the APMEN countries.

The greatest number of articles relate to the subjects of epidemiology and control, which reassuringly suggests that controlling and monitoring the spread of malaria remains a key priority. In contrast, the number of publications related to clinical trials is steadily decreasing, perhaps reflecting a global policy change to artemisinin combination therapy and the relative low priority given to drug monitoring. This development may be short sighted given the invariable emergence and spread of drug resistant strains of Plasmodia [10-12].

The study has potential limitations in selection bias and misclassification. Research publications are not always an accurate mirror of research activities. Highly relevant operative research may not be published but are of great value for programmes. Furthermore the extent to which an individual country is associated with a particular publication may vary widely. Publications that did not clearly distinguish whether the paper was from South Korea, North Korea or both, were attributed to both Koreas. This may have resulted in an inflated number of papers attributed to North Korea because there were only a small number of articles specifically relating to North Korea compared to those relating to South Korea. Also, assigning a paper to one country or subject is inappropriate when multiple countries or subjects are involved. Hence search algorithms targeting the title, keywords and abstract were used to identify and assign papers to multiple countries and subjects. This approach was found accurate in spot checks. Compared to manually extracting data from the text, using computer algorithms improves reproducibility. Also this methodology can be rapidly applied and replicated to maintain ongoing surveillance of temporal trends in publication outputs. Lastly, papers that did not mention a specific *Plasmodium *species could not be assigned to either *P. vivax *or *P. falciparum*.

Despite the challenges, 79 countries eliminated malaria between 1945 and 2010. Of the remaining 99 endemic countries, about one third have reduced the burden of malaria to low levels, and are now working towards malaria elimination [13]. The methods required for the elimination of malaria in the 20^th ^century may no longer be feasible or appropriate in the 21^st ^Century. The social structures of the last century have been transformed. No longer can divisions of health care workers be commandeered in a top down approach. Parasite and vector resistance has rendered many insecticides and antimalarial drugs useless. There is an urgent need for basic research to discover safe and effective interventions to prevent and cure all forms of malaria. There is also an urgent need for operational research to assist program managers in the optimization, scaling up, and monitoring of interventions. Further work is needed to identify funding, prioritize research topics, strengthen capacity in endemic countries, and facilitate multi-country and institution collaborations such as APMEN, that have the potential to evaluate new interventions across a range of endemic environments.

## Competing interests

The authors declare that they have no competing interests.

## Authors' contributions

FA searched the databases, analysed the data and contributed to the drafting of the manuscript. NMD provided advice regarding the analysis and contributed to the drafting of the manuscript. DB, GG, GQ, MSH, RK, KM, GT, and MW contributed to the interpretation of data and the drafting of the manuscript. RNP conceived of the study, and participated in its design and drafting of the manuscript. LvS participated in the design, contributed in the analysis and the drafting of the manuscript. All authors read and approved the final manuscript.

## Supplementary Material

Additional file 1 country search algorithm Click here for file

Additional file 2 study-type search algorithm Click here for file

Additional file 3 subject search algorithm Click here for file
