# Supplementary material for: Trends in malaria research in 11 Asian Pacific countries: an analysis of peer-reviewed publications over two decades
Source: Malar J. 2011 May 18;10:131. doi: 10.1186/1475-2875-10-131 (PMC3118956; doi:10.1186/1475-2875-10-131)
Supplement: Additional file 1 — country search algorithm [file 1475-2875-10-131-S1.DOC]

Additional file 1:

*Search Methods for acquiring article references*

We used the following search string: *Country* AND (vivax OR *malaria * OR *Plasmodium* or “*P. ovale*” OR “*plasmodium ovale*” OR *falciparum* OR *malaria* OR *knowlesi*) to search the MEDLINE database through Pubmed. The term “Country” was adapted to each of the APMEN countries. For example, the phrase used for Bhutan was “(Bhutan*[TIAB] OR Bhutan*[MH])”. The asterisk indicates a wild card and allows for extensions of the word Bhutan such as Bhutanese, and the “[TIAB]” and “[MH]” limits the search of these keywords to Title/Abstract and Mesh Terms respectively. The search terms for individual countries are shown in supplementary table S 1. The references were exported from Pubmed into Endnote X3 (Thompson Reuters, San Francisco, USA), duplicates were removed and the file was transferred to Excel 2007 (Microsoft Corp., Seattle, USA).

The articles retrieved using the search phrase ‘Korea’ were categorized as relating to the northern Democratic People's Republic of Korea (DPRK) or the southern Republic of Korea (ROK) depending on whether the terms DPRK, ROK, North Korea or South Korea were mentioned in the title, abstract or keywords. Any articles relating to Korea but not specifically North or South were included in both categories.

Country search algorithm

| **Country** | **Search Phrase** |
| --- | --- |
| Bhutan | (Bhutan*[TIAB] OR Bhutan*[MH]) |
| China | (China*[TIAB] OR China*[MH] OR Chinese*[TIAB] OR Chinese*[MH]) |
| Korea | (Korea*[TIAB] OR Korea*[MH] Or DPRK[TIAB] OR DPRK[MH] OR ROK[TIAB] OR ROK[MH]) |
| Indonesia | (Indonesia*[TIAB] OR Indonesia*[MH]) |
| Malaysia | (Malaysia*[TIAB] OR Malaysia*[MH]) |
| Philippines | (Philippine*[TIAB] OR Philippine*[MH]) |
| Solomon Islands | (“Solomon Island*”[TIAB] OR “Solomon Island*”[MH]) |
| Sri Lanka | (“Sri Lanka*”[TIAB] OR “Sri Lanka*”[MH]) |
| Thailand | (Thai*[TIAB] OR Thai*[MH]) |
| Vanuatu | (Vanuatu*[TIAB] OR Vanuatu*[MH]) |
