# Supplementary material for: Trends in malaria research in 11 Asian Pacific countries: an analysis of peer-reviewed publications over two decades
Source: Malar J. 2011 May 18;10:131. doi: 10.1186/1475-2875-10-131 (PMC3118956; doi:10.1186/1475-2875-10-131)
Supplement: Additional file 2 — study-type search algorithm [file 1475-2875-10-131-S2.DOC]

Additional file 2: Study-type search algorithm

| **Original** | **Derivative** | **Discarded** |
| --- | --- | --- |
| Comparative Study  Clinical Trial  Case Reports  Research Support  Evaluation Studies  Validation Studies  In Vitro  English Abstract  Letter | Review  Comment  Meta-Analysis  Editorial  News  Historical  Congresses | Biography |
