# Supplementary material for: Trends in malaria research in 11 Asian Pacific countries: an analysis of peer-reviewed publications over two decades
Source: Malar J. 2011 May 18;10:131. doi: 10.1186/1475-2875-10-131 (PMC3118956; doi:10.1186/1475-2875-10-131)
Supplement: Additional file 3 — subject search algorithm [file 1475-2875-10-131-S3.DOC]

Additional file 3: subject search algorithm

| **Subject** | Keywords | Syntax |
| --- | --- | --- |
| **Diagnostics** | Diagnosis  diagnostics | =IF(ISERROR(SEARCH("*diagnosis*",$M2,1)),"0","Diagnosis")  =IF(ISERROR(SEARCH("*diagnostics*",$M2,1)),"0","Diagnostics") |
| **Molecular/**  **Genetics** | genetics  Polymorphism | =IF(ISERROR(SEARCH("*genetic*",$M2,1)), IF(ISERROR(SEARCH("*polymorphism*",$M2,1)), "0","Polymorphism"),"Genetic") |
| **Clinical** | therapeutic use  aetiology  adverse effects  mortality | =IF(ISERROR(SEARCH("*therapeutic use*",$M2, 1)), IF(ISERROR(SEARCH("*etiology*",$M2,1)), IF(ISERROR(SEARCH("*adverse effects*",$M2, 1)),IF(ISERROR(SEARCH("*mortality*",$M2,1)), IF(ISERROR(SEARCH("*clinical*",$M2,1)), IF(ISERROR(SEARCH("*clinical*",$L2,1)),"0","Clinical (PT)"), "Clinical"),"Mortality"), "Adverse Effects"), "Etiology"),"Therapeutic Use") |
| **Entomology/**  **Insecticides** | Entomology  Insect  parasitology  Culicidae  Pyrethrins  Vector*  anopheles | =IF(ISERROR(SEARCH("*Entomology*",$M2, 1)), IF(ISERROR(SEARCH("*insect*", $M2,1)), IF(ISERROR(SEARCH("*parasitology*",$M2,1)), IF(ISERROR(SEARCH("*culicidae*",$M2,1)), IF(ISERROR(SEARCH("*pyrethrins*",$M2,1)), IF(ISERROR(SEARCH("*vector*",$M2,1)), IF(ISERROR(SEARCH("*anopheles*",$M2,1)),"0", "Anopheles"),"Vector"),"Pyrethrins"),"Culicidae"),"Parasitology"),"Insect"),"Entomology") |
| **Biology/**  **Biochemistry** | biology  Erythrocytes  enzymology  cytology  parasitology | =IF(ISERROR(SEARCH("*biology*",$M2, 1)), IF(ISERROR(SEARCH("*Erythrocytes*", $M2,1)), IF(ISERROR(SEARCH("*enzymology*",$M2,1)), IF(ISERROR(SEARCH("*cytology*",$M2,1)), IF(ISERROR(SEARCH("*parasitology*",$M2,1)),"0","Parasitology"),"cytology"),"enzymology"), "Erythrocytes"),"Biology") |
| **Social Sci/**  **Health Policy** | education  Health  Economics  Demography  ethnology | =IF(ISERROR(SEARCH("*education*",$M2, 1)), IF(ISERROR(SEARCH("*health*", $M2,1)), IF(ISERROR(SEARCH("*economics*",$M2,1)), IF(ISERROR(SEARCH("*demograph*",$M2,1)), IF(ISERROR(SEARCH("*ethnology*",$M2,1)),"0","Ethnology"),"Demography"),"Economics"),"Health"),"Education") |
| **Epidemiology/**  **Control** | epidemiology  isolation & purification  prevention & control | =IF(ISERROR(SEARCH("*epidemiology*",$M2, 1)), IF(ISERROR(SEARCH("*isolation & purification*", $M2,1)), IF(ISERROR(SEARCH("*prevention & control*",$M2,1)),"0", "prevention & control"),"isolation & purification"), "epidemiology") |
| **Immunology/**  **Vaccines** | Immun*  Antibodies  Vaccine | =IF(ISERROR(SEARCH("*immun*",$M2,1)), IF(ISERROR(SEARCH("*antibodies*",$M2,1)), IF(ISERROR(SEARCH("*vaccine*",$M2,1)), "0","Vaccine"),"Antibodies"),"Immun*") |
| **Pathophysiology** | physiology  physiopathology  Myometrium  Endometritis  Pathology  Parasitemia | =IF(ISERROR(SEARCH("*physio*",$M2, 1)), IF(ISERROR(SEARCH("*pathology*", $M2,1)), IF(ISERROR(SEARCH("*parasitemia*",$M2,1)),"0", "parasitemia"),"pathology"), "Physio") |
| **Drugs/**  **Drug Resistance** | Drug  Drug-administration Schedule  Chloroquine  Drug Combinations  Drug Resistance  drug effects  drug therapy  Medicine  administration & dosage  Dose-Response Relationship  pharmacology  pharmacokinetics  Antimalarials | =IF(ISERROR(SEARCH("*drug*",$M2, 1)), IF(ISERROR(SEARCH("*administration & dosage*", $M2,1)), IF(ISERROR(SEARCH("*pharmacokinetics*",$M2,1)), IF(ISERROR(SEARCH("*medicine*",$M2,1)), IF(ISERROR(SEARCH("*antimalarials*",$M2,1)), IF(ISERROR(SEARCH("*pharmacology*",$M2,1)), IF(ISERROR(SEARCH("*Dose-Response*",$M2,1)), "0", "Dose-Response"),"Pharmacology"), "Antimalarials"),"Medicine"),"pharmacokinetics"),"administration & dosage"), "Drug") |
